# Perceived Academic Support and Mental Well-Being Among Vocational Education and Training (TVET) Trainees in Kenya: The Mediating Role of Academic Resilience

**DOI:** 10.3390/ejihpe16060074

**Published:** 2026-05-25

**Authors:** Naomi Odira Owuor, Bettina F. Piko

**Affiliations:** 1 Doctoral School of Education, University of Szeged, 6722 Szeged, Hungary; naomi.odira.owuor@edu.u-szeged.hu; 2 Department of Behavioral Sciences, University of Szeged, 6725 Szeged, Hungary

**Keywords:** mental well-being, academic resilience, perceived academic support, technical and vocational training

## Abstract

Mental well-being has been considered a fundamental contributor to overall academic success and psychological stability. Based on the Transactional Theory of Stress and Coping, this study examined the mediating role of academic resilience in the relationship between perceived academic support and mental well-being of Kenyan TVET trainees. A quantitative cross-sectional design was employed, with a sample of 1933 trainees (*M*_age_ = 22.87 years; 57.7% male) from 239 public TVET institutions in Kenya. The following measures were administered: Perceived Academic Support Questionnaire, Academic Resilience Scale, and the Short Warwick–Edinburgh Mental Well-Being Scale. Correlation analysis demonstrated that perceived academic support showed a strong positive association with mental well-being, whereas academic resilience indicated a moderate association. Consistent with the hypothesized model, parallel mediation analysis indicated that academic resilience partially mediated the relationship between academic support and mental well-being. The indirect effects observed across resilience dimensions indicated that emotional response was the dominant mediating pathway, while perseverance showed a small positive indirect effect, and adaptive help-seeking demonstrated a small but significant negative indirect effect. These findings contribute to the growing but limited literature on well-being in vocational training and suggest that while academic resilience serves as a key mediating mechanism, perceived academic support may also function as a direct protective factor, underscoring the importance of embedding structured emotional coping support within TVET academic environments.

## 1. Introduction

Educational systems worldwide have recognized that mental health and well-being have emerged as central concerns for learning, academic success, and life satisfaction among students (Duncan et al., 2021; Duraku et al., 2024). Across all levels of learning, students have increasingly reported high levels of psychological distress, anxiety, emotional exhaustion, and depressive symptoms, all of which undermine their motivation and cognitive engagement (Alshammari et al., 2021; Brandseth et al., 2019; Gore, 2025). In the context of technical and vocational education and training (TVET), trainees are exposed to a compressed curriculum with intense hands-on training, practical-based assessment, and work–study-related instruction (Nefdt et al., 2025; Wen et al., 2024). Coupled with demands compounded by socio-economic inequalities, work-related demands, gendered course identities, and uncertain career trajectories, this learning environment is characterized not only by psychological strains but also by academic support and resilience (Achuodho et al., 2025; Fan & Liu, 2024). In Kenya, TVET training has historically been perceived as a pathway for academically lower-performing students from low-income families and rural areas with limited access to high-quality academic orientation, reinforcing a cultural stigma that associates vocational training with lower intellectual capability and social status (Opiyo et al., 2025; UNESCO, 2023). However, research has mainly focused on basic education and university settings, leaving TVET trainees underrepresented in mainstream educational and psychological research. As a result, addressing this gap is critical, as vocational pathways play a pivotal role in national workforce development, economic sustainability, and social mobility worldwide (UNESCO, 2023; Zhu et al., 2025).

From a psychological perspective, mental well-being is a relevant aspect of mental health, emphasizing positive mental functioning besides the absence of mental illness and negative mental states (e.g., extensive sadness). In other words, mental well-being provides important psychological resources and efficient functioning needed to maintain mental health (Gautam et al., 2024; Zábó et al., 2022). Mental well-being reflects positive psychological functioning such as life satisfaction, happiness, gratitude, and positive emotions (Martela, 2025; Solberg et al., 2023). According to the World Health Organization (2025), mental health is ‘a state of mental well-being that enables people to cope with the stresses of life, realize their abilities, learn well and work well, and contribute to their community’. This definition provides a holistic view of quality of life, purpose, and the ability to flourish and function optimally, including emotional balance, resilience, and sustained engagement in academic and social life (Arslan, 2023; Gautam et al., 2024; Kim & Lindeman, 2020). Considering mental well-being would facilitate an in-depth understanding and sophisticated awareness of how vocational training environments can enhance holistic growth, resilience, life satisfaction, and development (Norwich et al., 2022; Solberg et al., 2023). Given the effects of adverse academic demands and expectations of TVET curricula, it is important for stakeholders to develop policies and targeted interventions (such as academic support systems) to support the psychological well-being of trainees (Fu, 2024; Rana et al., 2023).

Building on the Transactional Theory of Stress and Coping (Lazarus & Folkman, 1984), this study views technical and vocational training as a psychologically demanding learning environment in which trainees’ well-being depends on how effectively they mobilize internal and external coping resources, either challenging or threatening (Qiang & Li, 2025). Lazarus and Folkman (1984) argue that trainees who appraise academic demands as challenges are more likely to utilize available coping resources effectively, sustaining psychological well-being, whereas those who appraise these demands as threats and perceive coping resources as insufficient are more vulnerable to distress. Within this framework, perceived academic support from teachers, peers, and parents represents a key external resource that strengthens trainees’ capacity to cope, whereas academic resilience reflects the internal adaptive mechanisms through which trainees manage academic challenges and sustain psychological functioning (Kvintová et al., 2025). Together, these external (perceived academic support) and internal (academic resilience) resources jointly interact within the appraisal process to shape trainees’ mental well-being outcomes. Therefore, this study investigates the mediating role of academic resilience in the link between perceived academic support and mental well-being.

### 1.1. Perceived Academic Support and Mental Well-Being

Perceived academic support is widely recognized as a key factor aligned with academic success and mental well-being, contributing to reduced depressive and anxiety symptoms (Fu, 2024; Pontes et al., 2024). It has been conceptualized as a subjective appraisal of the availability of supportive structures an individual receives from significant others, including teachers, parents, and peers (Pontes et al., 2024; Reyes et al., 2022; Ruihua et al., 2025a). This support is considered a vital psychological resource that shapes students’ academic experiences and interactions, fosters motivation, and supports cognitive development, psychological adjustment, and emotional balance while acting as a protective function against anxiety, depression, and loneliness (Çivitci, 2015; Jarrar & Nweke, 2025; Shafqat et al., 2024; O’Reilly et al., 2025; Villar et al., 2025; Zhu et al., 2025). Students with greater academic support report lower levels of distress, including symptoms of depression and self-harming behaviors (Gao et al., 2025). Mental health problems, such as academic burnout, depression, and suicidal tendencies, often arise from inadequate social support from peers, parents, and significant others (Addy et al., 2021; Li et al., 2025).

Within educational settings, academic support may be particularly salient due to the applied nature of training and the close interaction among parents, teachers, and peers, promoting a positive school environment (Lazarides et al., 2025; Solberg et al., 2023; Vansoeterstede et al., 2025). In collaboration with parental and peer support, trainers can implement actions for trainees’ mental well-being by adopting specific skills to acknowledge trainees’ challenges (Solberg et al., 2023). In the African context, particularly in Kenya, TVET trainees may experience limited academic support compared to their counterparts in tertiary institutions due to the stigma associated with technical training. Traditionally, these institutions have been perceived as having lower academic status, attracting trainees with lower academic capability. In addition, trainees are largely drawn from low-income families with limited access to educational resources, giving room for less prioritization of academic support structures, which may exacerbate mental well-being (Achuodho et al., 2025; Nefdt et al., 2025; Ruihua et al., 2025b; Opiyo et al., 2025). Within the transactional theory, perceived academic support constitutes a critical external coping resource that is expected to be positively associated with trainees’ mental well-being.

### 1.2. Academic Resilience and Mental Well-Being

Academic resilience in an educational context is regarded as an indicative characteristic of an individual’s academic success and well-being. Cassidy (2016) conceptualizes it as a construct that reflects an increased likelihood of academic success despite adversities. Likewise, Klutsey and Mahama (2025) refer to academic resilience as trainees’ capacity to adapt positively to academic challenges, maintain engagement, and recover from setbacks. Resilience skills play a critical role in trainees’ mental well-being, contributing to less emotional distress and greater academic motivation (Jianping et al., 2024; Yang et al., 2025). Resilient trainees are likely to appraise academic pressures as manageable challenges, applying coping resources that would shape their response to these pressures, reducing psychological strain.

As a result, academic resilience is strongly associated with lower symptoms of anxiety, depression, emotional exhaustion, and academic burnout (Ansari & Iqbal, 2025; G. D. Smith et al., 2025). Also, trainees tend to recover much faster from failures and uncertainties, maintaining academic adjustments and emotional balance (Cassidy, 2016). It also fosters positive mental well-being, greater life satisfaction, happiness, academic engagement, and accomplishment in studies through perseverance and motivation (Abbas et al., 2024; Klutsey & Mahama, 2025; Rana et al., 2023).

In TVET settings, where trainees are exposed to intensive academic workloads, hands-on training, and competence-based assessments, academic resilience may enable them to overcome academic constraints and achieve successful and fulfilling academic outcomes. Thus, it has a critical role in preventing psychological distress and promoting well-being (Abbas et al., 2024; Apriliana & Nalle, 2024; Yildirim et al., 2023), highlighting the importance of targeted resilience-building programs in vocational institutions as well as supportive structures in vocational environments (Yali & Binti Mohamad Nasri, 2025). Consistent with the transactional framework, academic resilience is considered an internal adaptive mechanism that enables trainees to reappraise academic stressors as manageable, thereby sustaining effective coping. Therefore, academic resilience is expected to be positively associated with both perceived academic support and mental well-being among TVET trainees.

### 1.3. Mediation Model of Academic Resilience

Academic resilience has been found to be one of the most important psychological constructs linking supportive structures to well-being (Fan & Liu, 2024; Hu et al., 2025; Melaku et al., 2025; Saleem & Zia, 2024). Premised on the transactional theory of stress and coping (Lazarus & Folkman, 1984), academic resilience is conceptualized as a pivotal adaptive mechanism through which academic supportive systems influence trainees’ mental stability, life satisfaction, and academic engagement (Scheffert & Henson, 2025). While teacher, peer, parental, and institutional supportive structures offer external resources that reduce academic strain and overwhelming emotions, they also strengthen trainees’ internal capacity to cope with and adapt to academic challenges (Kvintová et al., 2025). Thus, academic resilience can contribute to positive functions as well as boost academic success, linking academic support to mental well-being (Melaku et al., 2025; G. D. Smith et al., 2025). Grounded in this theoretical model, academic resilience is considered a protective construct for mental well-being. Therefore, it is expected to serve as a key mediating mechanism through which perceived academic support influences trainees’ mental well-being.

### 1.4. The Present Study

**Figure 1:**
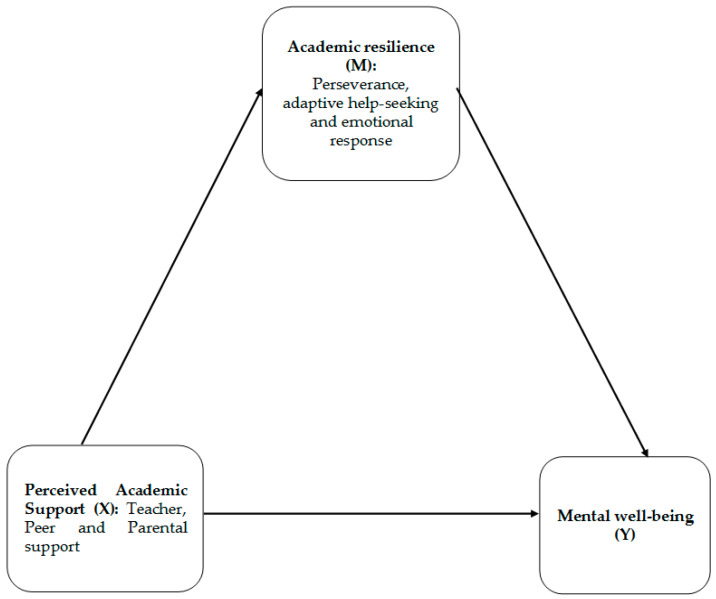
The hypothesized model showing the mediating role of academic resilience in the relationship between academic support and well-being.

In a vocational context, academic resilience dimensions, such as perseverance, would serve to enhance successful coping among trainees (Apriliana & Nalle, 2024; Schmid & Haukedal, 2022). However, despite growing evidence on the critical role in mediating the relationship between academic support and well-being, limited studies have examined these factors within the context of TVET in Africa. Therefore, this study may contribute to the existing literature in three specific ways. First, contextually, it addresses a significant gap by focusing on TVET trainees in Kenya, a population that is largely underrepresented in the mainstream psychological and educational research, despite the pivotal role of vocational pathways in national workforce development and social mobility (UNESCO, 2023). Second, theoretically, it applies the Transactional Theory of Stress and Coping (Lazarus & Folkman, 1984) to include perceived academic support as an external coping resource and academic resilience as an internal adaptive mechanism, providing a theoretically grounded explanation for how supportive structures translate into psychological well-being. Third, the analytical contribution lies in employing a parallel mediation design. This study considered a mediation model that moves beyond simple direct relationships to explicitly examine both direct and indirect effects across the three dimensions of perceived support on mental well-being among TVET trainees in Kenya. This analysis aims to clarify the mechanisms through which supportive structures may foster both academic functioning and psychological adaptation (Scheffert & Henson, 2025; G. D. Smith et al., 2025). Figure 1 illustrates the association between academic support and mental well-being and the mediating role of academic resilience in light of a concept that perceived academic support may promote psychological functioning and positive adjustments in vocational trainees.

The following research hypotheses were formulated for this study:

**Hypothesis** **1.** *There is a significant positive association between perceived academic support and mental well-being of TVET trainees (Alshammari et al., 2021; Shafqat et al., 2024).*

**Hypothesis** **2.** *There is a significant positive association between perceived academic support and academic resilience (Fan & Liu, 2024; Schmid & Haukedal, 2022).*

**Hypothesis** **3.** *There is a significant positive association between academic resilience and mental well-being of TVET trainees (G. D. Smith et al., 2025; Kvintová et al., 2025).*

**Hypothesis** **4.** *Academic resilience mediates the relationship between perceived academic support and mental well-being of TVET trainees (Melaku et al., 2025; Hu et al., 2025).*

## 2. Materials and Methods

### 2.1. Research Design and Participants

This study employed a quantitative cross-sectional survey design to investigate the mediating role of academic resilience in the relationship between perceived academic support and well-being among Kenyan TVET trainees across different age groups, study level, year of study, and course category at a single point in time, offering a snapshot of their experiences and perceptions regarding the study variables (Creswell & Creswell, 2018; Xueying et al., 2025). The study comprised 239 TVET institutions with a population of 402,000 across all regions in Kenya (Ministry of Education, Republic of Kenya, 2024). The inclusion criteria comprised participants who were registered in a public TVET institution in Kenya at the time of data collection and who were willing to participate voluntarily. In the case of trainees under 18 years, only those who had prior parental consent were included. Trainees from private TVET institutions were excluded from the study. Multistage cluster sampling techniques were used to select the TVETs and trainees, respectively. The institutions were grouped into geographical regions (clusters), namely, Nairobi, Mount Kenya, Coast, Western, and Rift Valley, and respondents were sampled from these designated groups (Hossan et al., 2023). Within each regional cluster, trainees were selected using simple random sampling to minimize bias and ensure equal selection probability, with proportional representation maintained across the five geographical regions of Kenya.

Given the online mode of administration, response tracking was limited; however, a total of 1933 questionnaires were returned fully completed and included in the final analysis. The sample of 1933 was established to allow for detecting small effect sizes and mediation pathways in a multivariate regression framework, substantially exceeding the minimum recommended for mediation analyses with multiple predictors (Fritz & MacKinnon, 2007). The participants were aged between 16 and 47 years (M = 22.87, SD = 3.35), where 1116 (57.7%) were male and 817 (42.3%) were female; 780 (40.4%) were enrolled in the first year, 699 (36.2%) in the second year, and 454 (23.5%) in the third year. Educational levels were distributed as Level 6 (33.7%), Level 5 (21.8%), Level 4 (25.9%), and Level 3 (18.6%), with a higher percentage of trainees from the technical (73.8%) category. Most participants (78.7%) registered low family income, while medium and high family income were reported at 17.8% and 3.4%, respectively. These demographics are summarized in Table 1.

### 2.2. Procedure

Data were collected from 1 to 30 November 2025 through a self-administered online questionnaire developed using Google Forms. Trainees accessed the questionnaire via a shareable link that was created and shared via digital platforms. An online consent form was provided to parents and guardians who had minors (<18 years) through the institutions’ communication channels (short message service). Only trainees whose parents registered consent accessed the questionnaire link and noted assent at the start of the questionnaire. It was noted that only a very small proportion of trainees across participating institutions were minors. Trainees read an introductory note with details of the study and are requested to provide informed consent at the beginning of the online questionnaire. Respondents were assured that their information would remain private and confidential and would be used only for the purpose of this study. The participants were also informed that they could withdraw from the study at any time without any consequences. To ensure data quality, the questionnaire was configured to require a response to each item before proceeding to the next, preventing incomplete submissions. The Google Form was also set to allow only one response per Google account, minimizing the possibility of duplicate entries. Ethical approval to conduct this research was granted.

### 2.3. Measures

#### 2.3.1. Independent Variable

The Perceived Academic Support Questionnaire (Reyes et al., 2022) was employed to assess the academic support perceived by trainees. This instrument includes a total of 12 items classified into three dimensions of support: family, teachers, and peers. Responses are ranked from 1 (strongly disagree) to 5 (strongly agree). Sample items include ‘My parents help me to make my education plans’, ‘In my college, there is a teacher who listens to me when I have something to say’, and ‘In my college, I have a friend who helps me when I have difficulties’. The scale displayed strong internal consistency, with an overall reliability of α = 0.95. It also exhibited high consistency for perceived teacher support (α = 0.82), perceived peer support (α = 0.83), and perceived parental support (α = 0.92).

#### 2.3.2. Dependent Variable

Mental well-being of trainees was measured using the Warwick–Edinburgh Mental Well-being Scale, developed and validated by Tennant et al. (2007) and O. R. F. Smith et al. (2017), respectively. The 14-item, five-point Likert scale ranges from 1 (none of the time) to 5 (all of the time). It elucidates positive functioning that enhances motivation and resilience among trainees. Examples of statements on this scale include ‘I’ve been dealing with problems well’, ‘I’ve been feeling confident’, ‘I’ve been able to make up my own mind about things’, and ‘I’ve been feeling cheerful’. The reliability value of this scale for the present study was α = 0.92.

#### 2.3.3. Mediating Variable

The Academic Resilience Scale (ARS-30), developed and validated by Cassidy (2016), was used to measure trainees’ cognitive, affective, and behavioral responses to academic adversities. ARS-30 is a 30-item, five-point Likert scale ranging from 1 (very likely) to 5 (very unlikely). It is a three-factor structure that assesses adaptive processes such as perseverance, reflective, and adaptive help-seeking, as well as negative affect and emotional response. Notably, the negative affect and emotional response dimension captures trainees’ capacity to actively regulate and manage negative emotional reactions to academic demands and not experiences of negative affect. This is reflected in items such as ‘I would stop myself from panicking’, whereby higher scores indicate greater emotional response capacity rather than greater affective distress. The scale is a multidimensional measure appropriate for vocational training trainees, as it captures the capacity to respond to academic demands. Sample items include ‘I would try to think of new solutions’, ‘I would feel like everything was ruined and was going wrong’, and ‘I would look forward to showing that I can improve my grades’. The scale displayed an internal consistency of α = 0.90, with the dimensions indicating perseverance (α = 0.77), reflective and adaptive help-seeking (α = 0.89), and negative affect and emotional response (α = 0.77).

#### 2.3.4. Validation of the Instrument

The three instruments employed in this study have demonstrated established validity across multiple cultural contexts and are broadly supported in the literature. The PASQ demonstrated good construct validity and adequate reliability across all three dimensions (Reyes et al., 2022), with further validation in African educational settings, confirming its cross-cultural applicability (Melaku et al., 2025). The ARS-30 demonstrated good internal reliability and construct validity across its three dimensions, consistent with the original reliability coefficients of α = 0.83 for perseverance, α = 0.78 for reflective and adaptive help-seeking, and α = 0.80 for negative affect and emotional response (Cassidy, 2016). The WEMWBS indicated strong construct validity in its original development, with a Cronbach’s α value of 0.89 in student samples and 0.91 in population samples (Tennant et al., 2007).

Even though this study did not perform confirmatory factor analysis, this decision was guided by two considerations. First, since Kenya is an English-speaking country, there was no need for translation of the instruments, eliminating the need for re-validation commonly associated with cross-cultural adaptation. Second, the internal consistency values obtained for the instruments; WEMWBS (α = 0.92), ARS-30 (α = 0.90), and PASQ (α = 0.95) are comparable to those of the original developers of the instruments. Therefore, this empirically supports the reliability and appropriateness of the instruments in use in the Kenyan TVET setting.

### 2.4. Data Analysis

Data were analyzed using the statistical software IBM SPSS version 26. Comprehensive statistical descriptive analyses were performed to characterize the demographics, perceived academic support, academic resilience, and mental well-being of the trainees by calculating means, standard deviation, and frequency distributions. Cronbach’s alpha was used for the reliability analysis performed to assess the internal consistency of scale items. Assumptions of normality, linearity, homoscedasticity, and multicollinearity were also tested before conducting inferential analyses. Normality was assessed through kurtosis statistics and inspection of frequency distributions; linearity and homoscedasticity were examined through residual plots. Multicollinearity was evaluated using VIF and tolerance values. The VIF values ranged from 1.19 to 3.41, which were well below the conventional threshold of 10, and tolerance values ranged from 0.29 to 0.84, which were all above the minimum threshold of 0.10, indicating that there were no multicollinearity concerns. Pearson’s product-moment correlation was implemented to examine bivariate associations between perceived academic support, academic resilience, and mental well-being.

PROCESS macro-4 (Hayes, 2022) was used for the mediation analysis to assess whether academic resilience mediated the relationship between perceived academic support and mental well-being among TVET trainees, using a bootstrapping procedure with 5000 samples and setting 95% confidence intervals and a significance level of *p* < 0.05 to test whether the indirect effects are significant. PROCESS Model 4 was selected since it is specifically designed for parallel multiple mediation, allowing multiple mediators to be examined simultaneously in a single model. The mediation analysis was conducted in two stages. In the first stage, an overall mediation model was estimated with the composite perceived academic support score as the predictor, the composite academic resilience score as the mediator, and mental well-being as the outcome variable. In the second stage, three separate parallel mediation models were estimated, one for each dimension of perceived academic support (teacher, peer, and parental support), with all three academic resilience dimensions (perseverance, adaptive help-seeking, and emotional response) entered simultaneously as mediators in each model. This approach allowed the examination of differential indirect effects of each academic resilience dimension while controlling for others.

## 3. Results

### 3.1. Descriptive Statistics and Correlation Analysis

First, descriptive statistics and correlation analysis were performed for all variables. Perceived academic support, academic resilience, and mental well-being indicated adequate variability across the sample (n = 1933), thus giving room for subsequent correlation and mediation analysis. Inspection of distributions and residual plots indicated no serious violations of normality and linearity assumptions. Second, bivariate correlations were conducted to examine the relationships among perceived academic support, academic resilience, and mental well-being. The overall trend of the descriptive and Pearson correlations analyses is summarized in Table 2.

Strong positive correlations were observed between perceived academic support subscales and mental well-being, teacher support (r = 0.68, *p* < 0.01), peer support (r = 0.67, *p* < 0.01), and parental support (r = 0.74, *p* < 0.01). Notably, strong correlations were detected among the three perceived academic support dimensions, with teacher support correlated with peer support (r = 0.79, *p* < 0.01) and parental support (r = 0.81, *p* < 0.01), while peer support strongly correlated with parental support (r = 0.78, *p* < 0.01). Perceived academic support dimensions showed positive correlations with academic resilience subscales. Perseverance was moderately associated with teacher support (r = 0.28, *p* < 0.01), peer support (r = 0.29, *p* < 0.01), and parental support (r = 0.34, *p* < 0.01). Adaptive help-seeking showed weak correlation with teacher support (r = 0.12, *p* < 0.01), peer support (r = 0.16, *p* < 0.01), and parental support (r = 0.18, *p* < 0.01), whereas emotional response indicated moderate correlation with teacher support (r = 0.46, *p* < 0.01), peer support (r = 0.46, *p* < 0.01), and parental support (r = 0.57, *p* < 0.01). Additionally, perseverance (r = 0.27, *p* < 0.01) and adaptive help-seeking (r = 0.10, *p* < 0.01) showed a weak correlation with mental well-being, whereas emotional response (r = 0.57, *p* < 0.01) was moderately correlated with mental well-being. Notably, the pattern of correlations suggests that among the resilience dimensions, emotional response demonstrates the strongest and most consistent associations with both perceived academic support and mental well-being. Overall, the correlation results supported hypotheses 1 to 3, indicating significant positive associations among perceived academic support, academic resilience, and mental well-being.

### 3.2. Mediation Analysis

**Figure 2:**
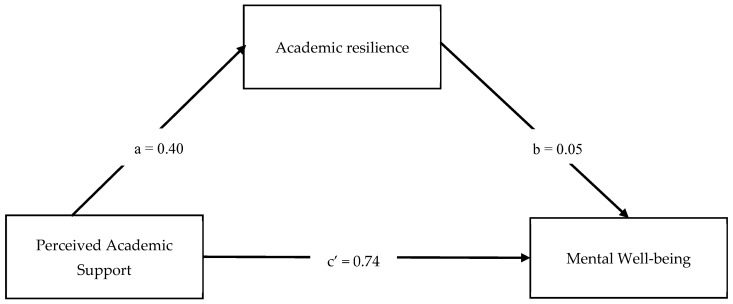
The final model showing the mediating role of academic resilience in the relationship between academic support and well-being.

The PROCESS macro-Model 4 (Hayes, 2022) with 5000 bootstrap samples was used to assess the mediating role of academic resilience in the relationships between perceived academic support and mental well-being. Parallel multiple mediation analyses tested whether academic resilience subscales mediated the relationships between the perceived academic support dimensions and mental well-being. Mediation analysis was conducted in two stages; the first phase was the overall mediation model, which tested the indirect effect of perceived academic support on mental well-being through academic resilience. Indirect effects were evaluated using 95% bootstrap confidence intervals, and effects were considered significant when the CI did not include zero. The mediation pathways were presented in an overall structural model (Figure 2), illustrating the interrelationships between perceived academic support, academic resilience, and mental well-being.

Table 3 presents the mediation analysis results indicating that the model was significant (F(2, 1930) = 1297.38, *p* < 0.001), with 57.3% of variance in mental well-being (R^2^ = 0.573). Perceived academic support had a significant direct effect on mental well-being (B = 8.36, β = 0.74, SE = 0.18, t = 45.28, *p* < 0.001) and significantly predicted academic resilience (B = 7.43, β = 0.40, SE = 0.38, t = 19.31, *p* < 0.001), which in turn had a small but significant effect on mental well-being (B = 0.03, β = 0.05, SE = 0.01, t = 3.14, *p* = 0.002). The indirect effect of perceived academic support on mental well-being through academic resilience was small but significant (B = 0.23, BootSE = 0.09, 95% CI [0.07, 0.40]). The completely standardized indirect effect was also significant (β = 0.02, BootSE = 0.01, 95% CI [0.01, 0.04]), indicating partial mediation and supporting hypothesis 4.

In the second phase, a parallel mediation model was conducted to examine the role of academic resilience subscales (perseverance, adaptive help-seeking, and emotional response) in the relationship between perceived academic support dimensions (parental, peer, and teacher support) and mental well-being. Teacher support significantly and positively predicted all three academic resilience mediators: perseverance (B = 0.76, SE = 0.06, t = 12.61, *p* < 0.001), adaptive help-seeking (B = 0.27, SE = 0.05, t = 5.16, *p* < 0.001), and emotional response (B = 0.80, SE = 0.03, t = 23.02, *p* < 0.001). In turn, emotional response was a strong positive predictor of mental well-being (B = 0.63, SE = 0.04, t = 16.97, *p* < 0.001), while adaptive help-seeking negatively predicted mental well-being (B = −0.10, SE = 0.04, t = −2.62, *p* = 0.009). Perseverance did not significantly predict mental well-being (B = 0.05, SE = 0.04, t = 1.52, *p* = 0.129). The direct effect of teacher support remained strong and significant after accounting for all mediators (B = 1.84, SE = 0.06, t = 30.67, *p* < 0.001). The bootstrapping results revealed a significant total indirect effect of teacher support on mental well-being (B = 0.52, BootSE = 0.04, 95% CI [0.44, 0.61]). Specifically, emotional response indicated a strong positive mediation effect (B = 0.51, BootSE = 0.04, 95% CI [0.43, 0.59]), whereas adaptive help-seeking indicated a small but significant negative indirect effect (B = −0.03, BootSE = 0.01, 95% CI [−0.05, −0.01]). Perseverance did not function as a significant mediator (B = 0.04, BootSE = 0.03, 95% CI [−0.01, 0.09]). The overall mediation model was statistically significant in approximately 55% of the variance on mental well-being (R^2^ = 0. 0.55, F(4, 1928) = 590.88, *p* < 0.001). Table 4 presents the bootstrapped indirect effect of teacher support on mental well-being through the dimensions of academic resilience.

From the model, peer support positively predicted mental well-being (B = 1.80, SE = 0.06, t = 29.265, *p* < 0.001, 95% CI [1.68, 1.92]), indicating a significant direct effect after controlling for the dimensions of academic resilience. Significant prediction through peer support were observed for perseverance (B = 0.80, SE = 0.06, t = 13.22, *p* < 0.001), adaptive help-seeking (B = 0.36, SE = 0.05, t = 6.96, *p* < 0.001), and emotional response (B = 0.80, SE = 0.03, t = 22.86, *p* < 0.001). In turn, perseverance (B = 0.09, SE = 0.04, t = 2.62, *p* = 0.009) and emotional response (B = 0.65, SE = 0.04, t = 17.00, *p* < 0.001) positively predicted mental well-being, while adaptive help-seeking (B = −0.17, SE = 0.04, t = −4.30, *p* < 0.001) negatively predicted well-being. As illustrated in Table 5, there was a significant positive mediation through perseverance (B = 0.08, BootSE = 0.03, 95% CI [0.02, 0.13]) and emotional response (B = 0.52, BootSE = 0.04, 95% CI [0.43, 0.61]), whereas adaptive help-seeking showed a negative significant effect (B = −0.06, BootSE = 0.02, 95% CI [−0.10, −0.03]). A significant total indirect effect on mental well-being through the academic resilience dimensions was observed (B = 0.53, BootSE = 0.05, 95% CI [0.45, 0.62]), indicating partial mediation.

The outcome of mediation analysis for perceived parental support, as illustrated in Table 6, indicated that perceived parental support significantly and positively predicted all three academic resilience mediators: perseverance (B = 0.49, SE = 0.03, t = 16.02, *p* < 0.001), adaptive help-seeking (B = 0.22, SE = 0.03, t = 8.10, *p* < 0.001), and emotional response (B = 0.51, SE = 0.02, t = 30.25, *p* < 0.001). In turn, emotional response (B = 0.45, SE = 0.04, t = 11.71, *p* < 0.001) positively predicted mental well-being, while adaptive help-seeking (B = −0.15, SE = 0.04, t = −3.93, *p* < 0.001) negatively predicted well-being. However, perseverance did not significantly (B = 0.06, SE = 0.03, t = 1.63, *p* = 0.104) predict it. The direct effect of parental support on mental well-being remained significant (B = 1.10, SE = 0.03, t = 33.82, *p* < 0.001, 95% CI [1.04, 1.17]), indicating partial mediation. The bootstrapping results revealed a significant total indirect effect of parental support on mental well-being through the dimensions of academic resilience (B = 0.22, BootSE = 0.03, 95% CI [0.18, 0.28]). Specifically, emotional response demonstrated a strong positive indirect effect (B = 0.23, BootSE = 0.03, 95% CI [0.18, 0.28]), and adaptive help-seeking demonstrated a small but significant negative indirect effect (B = −0.03, BootSE = 0.01, 95% CI [−0.05, −0.02]), whereas perseverance did not significantly mediate the relationship (B = 0.03, BootSE = 0.02, 95% CI [−0.01, 0.06]).

The overall mediation model explained 57.3% of the variance in mental well-being (R^2^ = 0.573, f^2^ = 1.342), representing a large effect size (Cohen, 1988). Similarly, the parallel mediation models demonstrated large effect sizes among the teacher support model (R^2^ = 0.55, f^2^ = 1.226), peer support model (R^2^ = 0.54, f^2^ = 1.161), and parental support model (R^2^ = 0.58, f^2^ = 1.383). Among the indirect effects, emotional response consistently demonstrated the strongest practical effect across all perceived support dimensions (0.23 ≤ *B* ≤ 0.52), whereas perseverance showed small positive and adaptive help-seeking showed small negative indirect effects.

## 4. Discussion

The purpose of this study was to investigate the mediating role of academic resilience in the relationship between perceived academic support and mental well-being among Kenyan TVET trainees. The findings strengthen how academic resilience serves as an indirect pathway linking academic support to mental health and well-being outcomes.

Perceived academic support was strongly associated with mental well-being in bivariate analysis. All subscales of perceived academic support exhibited a high degree of correlation with each other and well-being, with parental support having the strongest correlation (r = 0.74, *p* < 0.01). Prior studies have also indicated that perceived support is a critical resource shaping trainees’ academic experiences, promoting emotional balance and enhancing positive outcomes (Jarrar & Nweke, 2025; O’Reilly et al., 2025; Villar et al., 2025). In particular, parental support emerged as providing a strong protection for well-being. Consistent with the present study, the findings of Vansoeterstede et al. (2025) reported that parental attachment was negatively correlated with school burnout (r = −0.34, *p* < 0.001) and positively correlated with schoolwork engagement (r = 0.25, *p* < 0.001), confirming that parental supportive structures are closely linked to students’ academic well-being outcomes.

Academic resilience demonstrated moderate associations with perceived academic support and mental well-being, drawing its relevance as a construct that enables trainees to adapt positively to academic pressures and challenges and enhances their mental well-being. These findings align with the results of Yildirim et al. (2023), who found that resilience (β = 0.20, *p* < 0.01) and perceived support from family (β = 0.12, *p* < 0.05), friends (β = 0.14, *p* < 0.05), and significant others (β = 0.13, *p* < 0.05) significantly predicted positive emotional well-being among young adults. Among the dimensions of resilience, emotional response showed the strongest associations with academic support as well as with mental well-being, suggesting that trainees’ capacity to respond emotionally to academic demands is closely tied to supportive learning environments. This pattern is consistent with prior studies demonstrating that academic resilience is a critical psychological tool that reduces psychological distress, vulnerability, and academic burnout (Ansari & Iqbal, 2025) and fosters psychological functioning, such as a positive mindset and a sense of purpose and fulfillment (Jianping et al., 2024).

Parallel mediation analysis showed that the academic resilience dimensions, perseverance, adaptive help-seeking, and emotional response, significantly mediated the relationship between perceived academic support from teachers, peers, and parents and mental well-being. The results revealed that academic resilience dimensions partially mediated the relationship between academic support and mental well-being. These findings demonstrated that academic support is not only directly linked to mental well-being but also to the adaptive coping mechanism of academic resilience. Academic resilience, defined by Cassidy (2016) as a construct that reflects the ability to adapt through academic success despite adversities, enables trainees to utilize supportive structures to navigate academic demands and challenges. Empirical evidence supports these findings that academic support contributes to psychological functioning and well-being through adaptive mechanisms beyond resilience, reinforcing its role as a diverse protective resource within educational settings (Addy et al., 2021; Hu et al., 2025). In concordance with these findings, Melaku et al. (2025) confirmed that academic resilience fully mediated the relationship between perceived academic support and academic outcomes among Ethiopian university students, with perceived teacher support (β = 0.18, *p*  <  0.001) and perceived family support (β = 0.201, *p*  <  0.001) significantly predicting resilience and resilience in turn strongly predicting academic outcomes (β = 0.59, *p* < 0.001), supporting the mediating role of resilience as an adaptive pathway linking supportive structures to student outcomes.

Further to mediation models, academic resilience showed an indirect pathway, linking perceived academic support to mental well-being. This suggests that supportive academic structures may enhance trainees’ mental well-being by strengthening academic resilience and their capacity to cope with academic pressures. In other words, when trainees perceive greater support from teachers, peers, and parents, they are more likely to develop adaptive responses to challenges and better mental well-being outcomes. These findings are in concordance with prior studies (Melaku et al., 2025; Ruihua et al., 2025a; G. D. Smith et al., 2025). The mediation results demonstrated significant indirect effects of academic resilience across all its dimensions, indicating that perceived academic support was associated with mental well-being through trainees’ adaptive coping capacities.

Across all dimensions of academic resilience, emotional response emerged as the dominant pathway demonstrating the strongest positive indirect effect, whereas perseverance and adaptive help-seeking showed moderate to small positive and negative indirect effects, respectively. These patterns suggested that the benefits of supportive help perceived by trainees may foster their ability to manage emotional responses to academic challenges to greater heights and, to some extent, encourage perseverance and reliance on assistance (Hu et al., 2025; Klutsey & Mahama, 2025; Yali & Binti Mohamad Nasri, 2025). While adaptive help-seeking is generally conceptualized as a positive coping strategy that enables students to navigate academic demands (Cassidy, 2016; Klutsey & Mahama, 2025), its negative indirect effect may reflect the unique contextual dynamics of the Kenyan TVET environment. These results suggest that trainees who already feel stigmatized as academically less capable may perceive help-seeking as evidence of their academic inadequacy, thereby undermining their psychological well-being rather than enhancing it (Achuodho et al., 2025; Nefdt et al., 2025). Consistent with the findings of Ruihua et al. (2025b), students may perceive the need for help-seeking as a sign of academic weakness and a lack of independence.

### Strengths and Limitations

The findings of this study portray that academic resilience plays a critical psychological mechanism through which perceived academic support translates into improving mental well-being among TVET trainees in Kenya. In light of the unique challenges associated with technical and vocational training, the study contributes to the growing body of literature on the mediating role of academic resilience as a psychological resource through which supportive systems may foster trainees’ well-being (Melaku et al., 2025; G. D. Smith et al., 2025; Yildirim et al., 2023).

Despite these strengths, however, there are several limitations that need to be acknowledged. First, the nature of practical and competency-based training in TVET programs may limit the generalizability of the findings to other educational contexts. Future studies should consider comparative and longitudinal designs between students from TVET and general academic settings to strengthen the robustness of the findings. Second, the study relied on self-reported measures administered online, where trainees’ responses might be influenced by situational factors, recall inaccuracies, and participants’ subjective interpretation of items, thereby affecting the precision and the sensitivity of the measured psychological outcomes. Additionally, the exclusive reliance on self-report measures raises concerns about common method variance, whereby the shared measurement method may have artificially inflated the observed associations among study variables and potentially influenced the pattern of mediation results. Additionally, the administration of online questionnaires may have excluded trainees with limited access to internet connectivity or digital devices, potentially affecting the representativeness of the sample. Third, the cross-sectional design restricts causal inference. Therefore, the mediation findings should be interpreted as associative patterns rather than causal pathways. In the future, longitudinal research design will help clarify the temporal ordering of these variables and strengthen causal inference, while multilevel analyses incorporating institutional variables such as training climate and center resources will provide a more comprehensive understanding of the mechanisms involved. Finally, the high intercorrelations observed among the three perceived academic support dimensions (r = 0.78 to 0.81) suggest potential conceptual overlap, which may limit the distinctiveness of their individual contributions to the mediation models. Future studies should examine whether these dimensions represent distinct constructs or reflect a unified academic support factor. Additionally, while the instruments demonstrated adequate reliability and established validity across diverse cultural contexts, none of the three scales has been formally validated within a Kenyan TVET population, which represents a limitation that future studies should address through confirmatory factor analyses.

## 5. Conclusions

This study demonstrates that academic support from teachers, peers, and parents significantly influences mental well-being among TVET trainees through the mediating role of academic resilience, perseverance, emotional response, and adaptive help-seeking, thereby contributing important evidence to the limited non-Western literature. The persistence of the direct effect further suggests that perceived academic support operates as a multifaceted protective factor beyond academic resilience. Within the TVET context, where learning is characterized by industrial-aligned training and competence-based assessment, strengthening structured support systems may play a critical role in promoting psychological adjustment. These findings provide practical and operational implications. First, TVET institutions should pay more attention to embedding structured emotional coping mechanisms within academic support services, such as stress management workshops, peer support groups, guidance services, and awareness programs, to improve well-being. Second, resilience-focused training should be prioritized to provide optimal direction for TVET institutions’ psychological needs. Third, institutional policies should systematically integrate the psychological well-being principles into the TVET curriculum, particularly perseverance and emotional response. Lastly, given the negative indirect effect of adaptive help-seeking, TVET stakeholders should examine how help-seeking is culturally perceived within this environment and develop destigmatizing interventions that encourage trainees to seek support without evoking feelings of academic inadequacy.

## Figures and Tables

**Table 1:** The participants’ demographic characteristics.

Variable	Category	n	%
Age (Years)	M = 22.87, SD = 3.35 Range: 16–47	-	-
Gender	Male	1116	57.7
	Female	817	42.3
Year of Study	First Year	699	36.2
	Second Year	780	40.4
	Third Year	454	23.5
Level of Study	Level 3	360	18.6
	Level 4	500	25.9
	Level 5	422	21.8
	Level 6	651	33.7
Course Classification	Technical	1427	73.8
	Business	506	26.2
Family Income (Monthly)	Low (≤KSh 14,000)	1522	78.7
	Medium (KSh 14,001–56,000)	345	17.8
	High (≥KSh 56,000)	66	3.4

Notes. n = number of observations, M = mean; SD = standard deviation; KSh = Kenyan Shilling.

**Table 2:** Descriptive statistics and bivariate associations among study variables (n = 1933).

Variable	M	SD	1	2	3	4	5	6	7
1. Teacher Support	10.40	3.64	-						
2. Peer Support	10.50	3.59	0.79	-					
3. Parental Support	22.15	6.96	0.81	0.78	-				
4. Perseverance	49.57	9.96	0.28	0.29	0.34	-			
5. Adaptive Help-Seeking	33.68	8.35	0.12	0.16	0.18	0.79	-		
6. Emotional Response	22.88	6.28	0.46	0.46	0.57	0.43	0.21	-	
7. Mental Well-Being	46.81	12.54	0.68	0.67	0.74	0.27	0.10	0.57	-

Notes. M = mean; SD = standard deviation. *p* < 0.01 (two-tailed).

**Table 3:** The direct and total effect of perceived academic support on mental well-being through academic resilience.

Outcome	Predictor	B	β	SE	t	*p*	95% CI
Academic resilience	Perceived academic support	7.43	0.40	0.38	19.31	<0.001	[6.67, 8.18]
Mental well-being	Academic resilience	0.03	0.05	0.01	3.14	0.002	[0.01, 0.05]
Mental well-being	Perceived academic support (direct effect)	8.36	0.74	0.18	45.28	<0.001	[7.99, 8.72]
Mental well-being	Perceived academic support (total effect)	8.59	0.76	0.17	50.73	<0.001	[8.26, 8.92]

Note. B = unstandardized coefficient; β = standardized coefficient; SE = standard error; CI = confidence interval.

**Table 4:** Bootstrapped indirect effects of teacher support on mental well-being through academic resilience dimensions.

Predictor Path	B	BootSE	95% Boot CI
Teacher support → Perseverance → Mental well-being	0.04	0.03	[−0.01, 0.09]
Teacher support → Adaptive help-seeking → Mental well-being	−0.03	0.01	[−0.05, −0.01]
Teacher support → Emotional response → Mental well-being	0.51	0.04	[0.43, 0.59]
Total indirect effect	0.52	0.04	[0.44, 0.61]

Note. B = unstandardized coefficient; BootSE = bootstrapped standatd error; SE = standard error; CI = confidence interval.

**Table 5:** Bootstrapped indirect effects of peer support on mental well-being through academic resilience dimensions.

Predictor Path	B	BootSE	95% Boot CI
Peer support → Perseverance → Mental well-being	0.08	0.03	[0.02, 0.13]
Peer support → Adaptive help-seeking → Mental well-being	−0.06	0.02	[−0.10, −0.03]
Peer support → Emotional response → Mental well-being	0.52	0.04	[0.43, 0.61]
Total indirect effect	0.53	0.05	[0.45, 0.62]

Note. B = unstandardized coefficient; BootSE = bootstrapped standatd error; SE = standard error; CI = confidence interval.

**Table 6:** Bootstrapped indirect effects of parental support on mental well-being through academic resilience dimensions.

Indirect Path	B	BootSE	95% Boot CI
Parental support → Perseverance → Mental well-being	0.03	0.02	[−0.01, 0.06]
Parental support → Adaptive help-seeking → Mental well-being	−0.03	0.01	[−0.05, −0.02]
Parental support → Emotional response → Mental well-being	0.23	0.03	[0.18, 0.28]
Total indirect effect	0.22	0.03	[0.17, 0.28]

Note. B = unstandardized coefficient; BootSE = bootstrapped standatd error; SE = standard error; CI = confidence interval.

## Data Availability

The datasets generated during the current study are not publicly available due to privacy or ethical restrictions but are available from the corresponding author upon reasonable request.

## Abbreviations

The following abbreviations are used in this manuscript:

TVET	Technical and Vocational Education and Training
IBM	International Business Machines
SPSS	Statistical Package for Social Sciences
KSh	Kenyan Shilling
M	Mean
SD	Standard Deviation
SE	Standard Error
CI	Confidence Interval
